# Predictors of well-being and quality of life in men who underwent radical prostatectomy: longitudinal study^1^

**DOI:** 10.1590/1518-8345.2601.3031

**Published:** 2018-09-03

**Authors:** Adilson Edson Romanzini, Maria da Graça Pereira, Caroline Guilherme, Adauto José Cologna, Emilia Campos de Carvalho

**Affiliations:** 2 PhD.; 3 PhD, Associate Professor, Escola de Psicologia, Universidade do Minho, Braga, Portugal.; 4 PhD, Adjunct Professor, Curso de Enfermagem e Obstetrícia, Universidade Federal do Rio de Janeiro, Macaé, RJ, Brazil.; 5 PhD, Senior Professor, Faculdade de Medicina de Ribeirão Preto, Universidade de São Paulo, Ribeirão Preto, SP, Brazil.; 6 PhD, Senior Professor, Escola de Enfermagem de Ribeirão Preto, Universidade de São Paulo, PAHO/WHO Collaborating Centre for Nursing Research Development, Ribeirão Preto, SP, Brazil.

**Keywords:** Prostatectomy, Quality of Life, Well-Being, Medical-Surgical Nursing, Postoperative Care, Prostatic Neoplasms

## Abstract

**Objective:**

to identify socio-demographic, clinical and psychological predictors of well-being and quality of life in men who underwent radical prostatectomy, in a 360-day follow-up.

**Method:**

longitudinal study with 120 men who underwent radical prostatectomy. Questionnaires were used for characterization and clinical evaluation of the participant, as well as the instruments Visual Analog Scale for Pain, The Ways of Coping Questionnaire, Hospital Depression and Anxiety Scale, Satisfaction with Social Support Scale, Marital Satisfaction Scale, Subjective Well-Being Scale and Expanded Prostate Cancer Index. For data analysis, the linear mixed-effects model was used.

**Results:**

the socio-demographic factors age and race were not predictors of the dependent variables; time of surgery, problem-focused coping, and anxiety were predictors of subjective well-being; pain, anxiety and depression were negative predictors of quality of life; emotion-focused coping was a positive predictor. Marital dissatisfaction was a predictor of both variables.

**Conclusion:**

predictor variables found were different from the literature: desire for changes in marital relationship presented a positive association with quality of life and well-being; emotion-focused coping was a predictor of quality of life; and anxiety was a predictor of subjective well-being.

## Introduction

Prostate cancer, one of the most common neoplasms in the world^1^, is regarded as an obvious public health problem worldwide. It affects society by causing distress to patient’s and impacting economic aspects, and it requires substantial effort from health systems and professionals^2^.

The choice of the best treatment for localized prostate cancer depends on factors such as the risk of progression or death, urinary, sexual and intestinal functions, the patient’s preferences and well-being and quality of life prospects^1^. Radical prostatectomy (RP) is not free of complications, since intraoperative blood loss, lymphocele, infection, postoperative urinary incontinence, reoperation and erectile dysfunction may occur^3^ ^-^ ^4^. The suprapubic prostatectomy has an average duration of 02:47 hours^5^. 

Greater subjective well-being helps people generate more energy and be more active. Thus, it is a vital component for the recovery, treatment and quality of life of patients with prostate cancer^6^ ^-^ ^8^. This concept refers to the global cognitive assessment of individuals on their biological, psychological, sociocultural and spiritual aspects, and on how these feelings are experienced, which determines an affective component. This component, in turn, can be represented by positive or negative feelings based on individual standards and references, which results in the perception of satisfaction or dissatisfaction with life^9^ ^-^ ^11^. 

Health-related quality of life is considered as important as prostate cancer control itself, since changes in quality of life have been shown to affect satisfaction with the treatment outcome^12^. This concept is characterized as a feeling of satisfaction and prosperity in the context of the needs and capacities of the human being. However, the role of health-related quality of life for the selection of the systemic therapy for patients with prostate cancer remains uncertain^13^.

Studies indicate that factors related to the health of the individual and to the surgery^14^ ^-^ ^17^, in addition to socio-cultural, emotional and physical aspects^9^ ^,^ ^13^ and the conditions for the performance of daily life activities^18^, determine well-being and quality of life prospects for the surgical recovery of patients submitted to radical prostatectomy. The parameter considered is conditions superior or equivalent to those of the preoperative period. 

Factors such as increased age^19^ ^-^ ^20^, longer time of surgery and prolonged exposure to the anesthetic procedure and anesthetic agents^21^, complications in the patient recovery process^22^ ^-^ ^23^ pain after radical prostatectomy^24^ ^-^ ^26^, and unfavorable results regarding sexual function^27^ affected the patients’ perceptions of well-being and quality of life. 

Regarding the psychological factors, high capacity to cope with stress resulted in a lower intensity of the postoperative symptoms. Patients with lower capacity to cope with stress presented greater problems during surgery recovery^28^. Problem-focused coping was a positive predictor for psychological well-being and quality of life, while emotion-focused coping was negative^29^ ^-^ ^30^. 

Social support had positive effects on human life during difficult times, on recovery activities, well-being, health and adjustment to stress, which resulted in a better quality of life^31^ ^-^ ^33^. Psychological symptoms such as anxiety and depression were related to lower quality of life and well-being, with increased pain and sensitivity to symptoms. These symptoms may negatively influence patients’ motivation, energy, their coping with the disease, adherence to treatment and the recovery process^34^ ^-^ ^36^. Likewise, marital support was related to higher levels of quality of life, physical and mental health and recovery after radical prostatectomy^37^ ^-^ ^39^.

Understanding the surgical recovery of men after prostatectomy may favor the use of approaches directed to their characteristics. In this sense, the objective of this study was to identify socio-demographic, clinical and psychological predictive factors for the well-being and quality of life of men submitted to radical prostatectomy, in a 360-days follow-up.

## Method 

This is a longitudinal descriptive observational study^40^, conducted in the Urology Division of a public teaching hospital in the state of São Paulo, a reference in urologic oncology. Participants were men undergoing prostatectomy. After the medical indication for surgery, they were invited to the study by the main investigator. Those who agreed to participate in the study by signing the Informed Consent Term, had their data collected, respecting the dynamics of outpatient care and without any harm to medical treatment. 

Inclusion in the research occurred consecutively and the participants were followed up for a period of up to 360 days (T0 = baseline or preoperative, T1 = 30 days, T2 = 90 days, T3 = 180 days, T4 = 360 days post-operative follow-up). The follow-up window for applying the instruments varied, respecting the schedule established for medical care: T1 comprised data collection with a mean of 15.9 days (SD=7.2); T2 with mean of 91.4 days (SD=21.7); T3 with a mean of 203 days (SD=46.3); and T4 with a mean of 322.7 days (SD=48.6) after surgery. Regarding the collection process, there was a 6.5% to 12.2% loss to follow-up in the different periods.

The data collection in T0 occurred in the hospitalization unit and, in the other periods, it occurred in the outpatient sector. The presence or not of companions or caregivers in the room was at the discretion of the participant. 

The researcher assessed the participant’s ability to understand and respond to items of the instruments. For this, questions such as “What is the current date? What is the reason for hospitalization? What is the date and time of the surgery?” were asked. Then, the participants analyzed the instruments for their ability to respond to the items presented. 

Men with prostate cancer (stage T1-T3), selected for surgical treatment (RP) by the medical team, who did not present clinical signs of metastases, aged 18 years or older and who reported they were able to read and write in Portuguese were included in the research. Patients with a previous history of bladder or prostate surgery, diagnosis of neurological disease with probable repercussion on urinary control (for example, Parkinson’s disease, psychiatric disease, Alzheimer’s disease and spinal cord diseases) and those previously submitted to chemotherapy or radiotherapy were excluded.

The researcher approached 125 men who had clinical indication for prostate surgery. Of these, two did not meet the criteria (one had undergone chemotherapy and another had a prior surgery) and another three had the indication of surgery suspended. Data from 120 men undergoing prostatectomy were observed. 

In this research, there was no interference of the researcher in the treatment and no assistance provided to the patient. If necessary, the patient would be directed to the responsible multidisciplinary team, but there was no need for this procedure. 

The data collection instruments were completed with the researcher reading the instructions and the items. The instrusubment application time was approximately 40 minutes. 

For the characterization of the participants, the variables age, race/skin color, type of surgery, time of surgery, type of anesthesia, duration of anesthesia and ASA score were considered. For the clinical evaluation in the early postoperative period (T1), the variables duration of urinary catheter use and presence of complications were considered. In addition, seven instruments were used in the follow-up (T0-T4):

- *Visual Analog Scale for Pain (VAS)* - a one-dimensional self-reporting scale used to evaluate pain intensity in a 10-centimeter line, with “no pain” and “worst pain imaginable” at the extremities and “moderate pain” in the middle”^41^ ^-^ ^42^;
- *The Ways of Coping Questionnaire* ^43^ ^)^ - instrument adapted to the Brazilian culture^44^, with 66 items divided in 8 factors, answered on a Likert scale, with four possibilities: 0) Not at all, 1) A little, 2) pretty much, 3) a lot. In the factorial analysis carried out in the adaptation to Brazilian culture^44^, eight factors were identified (confrontation, distancing, self-controlling, social support, accepting responsibility, escape/avoidance, problem solving and positive reappraisal), and most items found in each factor presented a factorial load similar to those obtained by the authors of the instrument^43^. In the present study, all the items of the original scale were included, as in other studies^45^ ^-^ ^46^, and the eight classification factors initially proposed by the authors of the instrument were adopted^43^, but composed of the items indicated by the authors who adapted the instrument for Brazilian culture^44^. The ways of coping were classified into two categories: problem-focused coping and emotion-focused coping. The first is a combination of four-factors (confrontation, seeking social support, problem solving, and positive reappraisal), and the second is a combination of three-factors: distancing, accepting responsibility, and escape/avoidance. The factor self-controlling is considered independent, since it scores equally in both categories^47^ ^-^ ^48^. Higher scores in the instrument indicate greater coping capacity^43^ ^,^ ^49^. In this research, problem-focused coping presented Cronbach’s alpha of 0.87 and the emotion-focused coping presented Cronbach’ s alpha of 0.85;
- *Hospital Anxiety and Depression Scale (HADS)* ^50^ ^)^ - an instrument adapted for the Brazilian population^51^, with 14 multiple choice questions, consisting of two subscales: anxiety (HADS-A) and depression (HADS-D), with seven items in each. The score of each item ranges from zero to three, ant the total score in each subscale ranges from zero to 21. Results between 0 and 7 are considered normal, scores from 8 to 10 suggest the possibility of abnomarlity and more than 11 indicate probable abnormality. Score 8 is considered the cut-off point between the presence or absence of symptomatology^50^ ^-^ ^51^. In this study, the HADS score obtained a total Cronbach score of 0.71;
- *Satisfaction with Social Support Scale (SSSS)* ^52^ - this scale consists of 15 statements regarding the perception of support received from friends, family and community. They are distributed in four factors, and 6 items must be reverted for analysis. It is a 5-point Likert scale (5 - Totally agree, 4 - Partially agree, 3 - Neither agree nor disagree, 2 - Partially disagree and 1 - Strongly disagree), and the higher the score obtained, the greater the satisfaction with social support^52^. In this study, the scale presented Cronbach’s alpha of 0.77;
- *Marital Satisfaction Scale -* the instrument was validated for the Brazilian population^53^. There are three options for answering each item, which allow to qualify the level of satisfaction of the individual with respect to the conjugal aspects: 1) I like how it has been, 2) I would like it to be a little different, 3) I would like it to be a lot different. Thus, the higher the scores, the worse the results regarding marital satisfaction. This scale is composed of 24 items distributed in three domains of the conjugal union, each corresponding to a subscale: (a) satisfaction with the marital interaction, (b) satisfaction with the emotional aspects of the spouse, and (c) structural aspects, satisfaction with the form of organization and establishment and compliance of rules by the spouse. In this study, the scale presented Cronbach’s alpha of 0.95;
- *Subjective Well-Being Scale (SWBS)* ^12^ *-* this scale was constructed and validated for the Brazilian population and contains two subscales. The first one is composed of 54 items addressing feelings, emotions and evaluates the dimension of affection (positive and negative) that constitutes well-being. The person responds how he/she has felt lately, in which 1 means not at all, 2 a little, 3 moderately, 4 quite a lot and 5 extremely. The second subscale is composed of 15 sentences that seek to represent satisfaction with life. The individual responds in a scale in which 1 means totally disagree, 2 disagree, 3 do not know, 4 agree and 5 fully agree. The higher the score, the better the subjective well-being. In this study, the alpha presented was 0.93;
- *Expanded Prostate Cancer Index (EPIC) -* an instrument that evaluates the quality of life (functions and discomfort) of the patient after treatment of prostate cancer^54^. It includes 50 questions, from four domains: urinary, which is subdivided into four subscales (Function, Discomfort, Incontinence and Irritation/Obstruction); intestinal, which is subdivided into two subscales (Function and Discomfort); sexual, which is subdivided into two subscales (Function and Discomfort); and hormonal, which is subdivided into two subscales (Function and Discomfort). The response options for each item of the EPIC are on the form of a 5-point Likert scale. The scores obtained are transformed into a scale of 0-100, with higher scores representing a better health-related quality of life^54^ ^-^ ^55^.

Regarding the data analysis, the results obtained in the continuous or discrete quantitative variables were described by measures of central tendency (mean) and by the respective measures of dispersion (standard deviation). The results of the categorical variables were described by their absolute values ​​or percentages. 

In order to evaluate whether the socio-demographic variables, intraoperative conditions and clinical and psychological variables were predictors of well-being and quality of life in the periods studied, the regression analysis method was used. Therefore, the linear mixed-effects model or random-effects model (Generalized Linear Mixed Models) was used^56^. This method allows to describe the temporal trend taking into account the correlation between successive means and to estimate the variation in basal measurement and rate of change over time.

The dependent variables in the study were the total scores of the SWB and EPIC scales, the total scores of the HADS domains (anxiety and depression), totals of the Ways of Coping Questionnaire domains (Problem-Focused Coping and Emotion-Focused Coping), the totals of the other scales of the study (VAS, Scale of Satisfaction with Social Support, Marital Satisfaction Scale), as well as socio-demographic (age and race) and clinical variables (duration of anesthesia and time of surgery). The Kolmogorov-Smirnov test was conducted in order to obtain a distribution for the response variables, and adherence was tested with the Gamma distribution. Thus, it was verified that for the SWB scale adequacy occurred at all times analyzed whereas for the EPIC rejection occurred only in the T0 period. 

In order to identify the best functional form, a local polynomial fit (non-parametric ‘loess’ method) was applied. In the first adjustments of the regression models, the model was tested with all the variables and the inclusion of the quadratic polynomial terms for the variables with polynomial fit. Subsequently, the variables that did not have statistical significance were manually removed. After their removel, a new adjustment was made with the remaining variables. This was done until only significant variables remained.

For all adjustments and tests performed, the significance level of 5% (alpha=0.05) was adopted and the program used was R version 3.3.0. The mixed-effect models analyze were performed using the MASS libraries (function ‘glmmPQL’) and ggplot2 for the elaboration of the figures.

## Results 

**Figura 1:**
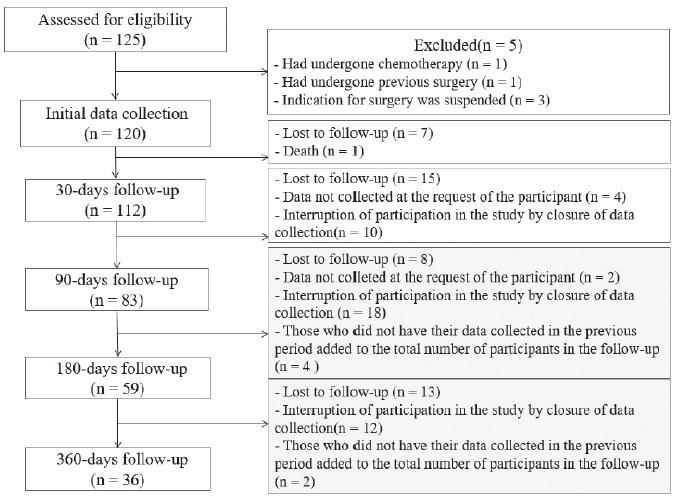
Flowchart of participants in the research in the different periods of data collection Ribeirão Preto, SP, Brazil, 2016

The initial number of participants (T0) in the study was 120 (Figure 1).

Regarding the socio-demographic, clinical and psychological variables of the participants, the mean age at the first observation was 63.8 (SD=7.7) years, the mean number of children was 3.1 (SD=2.0) and the educational level was 5.1 (SD=3.7) years. The majority (59.1%) were white, married/in a stable union (89.1%), retired (61.6%) and lived in the urban area (91.6%).

The mean time of surgery was 3hrs 57min (SD= 1 hr) and the mean duration of anesthesia was 4hrs 44min (SD = 01hr 15min). There was a predominance of balanced general anesthesia (62.8%), suprapubic prostatectomy surgery (97.4%) and patients classified as ASA 2 (79.5%), that is, mild systemic disease. The duration of urinary catheter use ranged from 11 to 48 days (mean = 14.7, SD = 5.5). In T1, 92.8% of the participants did not report complications or irregularities. The complications present were urinary tract infection (n = 2), fistula (n = 2), dehiscence and paresis of lower limbs (n = 1), and an unscheduled removal of urinary catheter. In addition, 96.4% had a clean and dry surgical incision. 

The mean scores of the other variables, in the T0-T4 periods, are listed below (Table 1).

**Table 1:** Distribution of the variables Pain, Coping, Psychological Morbidity, Satisfaction with Social Support, Marital Satisfaction, Subjective Well-Being and Quality of Life of men undergoing prostatectomy in the periods studied. Ribeirão Preto, SP, Brazil, 2016

Variable	T0*	T1^†^	T2^‡^	T3^§^	T4^||^
	M^¶^ (SD**)	M^¶^ (SD**)	M^¶^ (SD**)	M^¶^ (SD**)	M^¶^ (SD**)
Pain	120	112	83	59	36
	0.6(1.67)	2.4(2.7)	1.7(2.5)	1.0(2.1)	0.8(1.6)
Problem-focused coping	120	112	83	59	36
	1.6(0.5)	1.5(0.6)	1.6(0.6)	1.5(0.6)	1.2(0.7)
Emotion-focused coping	120	112	83	59	36
	1.2(0.6)	1.2(0.7)	1.3(0.7)	1.2(0.8)	1.4(0.6)
Psychological Morbidity	120	112	83	59	36
	10.1(5.7)	8.6(5.3)	9.2(6.5)	8.3(6.0)	8.8(6.2)
Anxiety Score	120	112	83	59	36
	6.0(3.5)	5.0(3.5)	5.0(3.5)	4.4(3.6)	4.6(3.5)
Depression Score	120	112	83	59	36
	4.1(3.2)	3.5(2.7)	4.2(3.7)	3.8(3.1)	4.1(3.3)
Satisfaction with Social Support	120	112	83	59	36
	3.8(0.6)	3.8(0.5)	3.8(0.5)	3.9(0.6)	3.8(0.6)
Marital Satisfaction	108	102	71	59	36
	1.7(0.5)	1.7(0.5)	1.8(0.6)	1.7(0.6)	1.9(0.5)
Subjective Well-Being	120	110	83	59	36
	2.7(0.5)	2.7(0.5)	2.6(0.4)	2.6(0.4)	2.5(0.5)
Quality of life	120	110	83	59	36
	81.8(11.1)	70.2(8.6)	68.6(9.5)	69.6(12.0)	74.5(11.7)
Urinary Function	120	110	83	59	36
	89.5(13.4)	75.2(15.4)	69.7(16.6)	77.6(18.9)	83.1(14.1)
Intestinal Habits	120	110	83	59	36
	92.2(10.9)	88.6(9.9)	92.5(9.7)	92.1(11.7)	94.4(11.9)
Sexual Function	120	110	83	59	36
	57.0(22.6)	29.0(12.4)	23.9(17.8)	23.4(19.4)	32.9(24.9)
Hormonal Function	120	110	83	59	36
	89.2(14.6)	90.2(12.4)	90.1(11.3)	86.7(17.7)	89.1(12.0)

*T0 - baseline; †T1 - 30 days; ‡T2 - 90 days; §T3 -180 days; ||T4- 360 post-operative days; ¶ M - mean; ** (SD) - standard deviation.

In the initial regression model, age, race, duration of anesthesia, pain, emotion-focused coping, depression and satisfaction with social support were not predictors of subjective well-being (p>0.05). In the final model of regression analysis, the variables time of surgery (p?0.000), problem-focused coping (p?0.000), anxiety (p=0.007) and marital satisfaction (p=0.010) were predictors of subjective well-being (Table 2). 

**Table 2:** Analysis of predictors of subjective well-being using linear mixed-methods models. Ribeirão Preto, SP, Brasil, 2016

Fixed Effects	Initial Model			Final Model			95% CI^†^
	β*	Standard error	p-value	β*	Standard error	p-value	
(Intercept)	0.713	0.216	0.001	0.810	0.036	0.000	2.094-2.414
Age	0.000	0.001	0.913				
Black Ethnicity	0.022	0.036	0.548				
Mixed Ethnicity	-0.002	0.037	0.950				
Time of Surgery	-0.191	0.732	0.794	0.183	0.260	0.482	0.721-1.999
Time of Surgery 2^‡^	0.573	0.032	0.030	0.865	0.253	0.000	1.444-3.907
Duration of anesthesia	0.027	0.032	0.390				
Pain	-0.002	0.003	0.413				
Problem-focused coping	0.053	0.025	0.038	0.058	0.014	0.000	1.028-1.090
Emotion-focused coping	0.004	0.023	0.843				
Anxiety	0.006	0.002	0.022	0.006	0.002	0.007	1.001-1.011
Depression	0.001	0.003	0.728				
Satisfaction with Social Support	-0.013	0.015	0.387				
Marital Satisfaction	0.037	0.015	0.015	0.037	0.014	0.010	1.009-1.068

*β - beta; †CI - confidence interval; ‡2 - Quadratic order polynomial terms.

It is expected that, for each one-point increase in problem-focused coping, there will be a relative increase of 5.9% in the mean of subjective well-being. For each one-point increase in the anxiety score, a relative increase of 0.6% in the mean well-being is expected. For each one-point increase in the marital satisfaction score, a relative increase of 3.8% in the mean well-being is expected, suggesting that the more dissatisfied one is with the marital relationship the greater their subjective well-being. The participants of this research did not present different means of well-being in the different periods analyzed, when compared with T0.

Regarding socio-demographic, psychological and clinical variables, pain (p?0.000), emotion-focused coping (p=0.013), anxiety (p=0.004), depression (p=0.009) and marital satisfaction (p=0.018) were predictors of quality of life (Table 3).

**Table 3:** Analysis of predictors of quality of life using linear mixed-methods models. Ribeirão Preto, SP, Brazil, 2016

Fixed Effects	Initial Model			Final Model			95% CI^†^
	β*	Standard Error	p-value	β*	Standard Error	p-value	
(Intercept)	4.369	0.118	0.000	4.441	0.016	0.000	82.175-87.679
Age	0.000	0.001	0.886				
Black Ethnicity	-0.017	0.028	0.537				
Mixed Ethnicity	0.006	0.028	0.814				
Time of Surgery	0.028	0.029	0.338				
Duration of Anesthesia	-0.022	0.024	0.371				
Pain	-0.013	0.003	0.000	-0.014	0.002	0.000	0.979-0.991
Problem-focused Coping	0.272	0.254	0.284				
Problem-focused Coping 2^‡^	0.023	0.186	0.898				
Emotion-focused Coping	-0.319	0.273	0.243	-0.149	0.155	0.336	0.635-1.167
Emotion-focused Coping 2^‡^	0.399	0.187	0.033	0.341	0.136	0.013	1.076-1.839
Anxiety	-0.513	0.168	0.002	-0.472	0.164	0.004	0.452-0.860
Anxiety 2^‡^	-0.436	0.130	0.001	-0.425	0.132	0.001	0.503-0.847
Depression	-0.005	0.002	0.047	-0.006	0.002	0.009	0.988-0.998
Satisfaction with Social Support	0.010	0.013	0.432				
Marital Satisfaction	-0.054	0.148	0.712	-0.061	0.147	0.676	0.703-1.255
Marital Satisfaction 2^‡^	0.294	0.140	0.037	0.330	0.139	0.018	1.058-1.829

*β - beta; †CI - confidence interval; ‡2 - Quadratic order polynomial terms.

For each one-point increase in the pain score, there is a relative reduction of 1.4% in the quality of life score, and for each one-point increase in the depression score, there is a relative reduction of 0.6% in the quality of life score.

When compared with T0, quality of life was lower in all postoperative periods (p <0.05). Therefore, the relative reduction expected in quality of life scores in relation to T0 is of 12.6% in T1, 15.9% in T2, 16.03% in T3 and 7.5% in T4. 

## Discussion

The literature reports frequent occurrence of imbalance or inequality in the number of participants in longitudinal studies^56^. In the present research, there was variation in the number of participants in the evaluation periods. Loss to follow-up may impair the internal validity and completion of the study^57^, but a participant’s withdrawal may be reversible. Thus, considering a single episode of non-response as non-participation may be premature^58^. This means that the analyzes may include temporary losses in previous moments, as occurred in this research in T2 and T3 (Figure 1). In order to adjust the data to the characteristics of the study design, analyzes were performed through mixed-effects models, which accept that the measurements of individuals do not need to be equal at all times^56^.

Regarding socio-demographic variables, age and race/color were not predictors, which was also found in other studies^55^ ^,^ ^59^. However, studies indicate that age greater than 60 years had greater impacts on quality of life^60^ and that white individuals had better survival rates when compared to blacks^61^ ^-^ ^62^.

Regarding the conditions of the surgical procedure, in the present study, time of surgery was a predictor of subjective well-being. There are reports in the literature that longer surgeries of radical prostatectomy are associated with more complications, longer periods of hospitalization and higher costs, which undermines the patient’s well-being^21^ ^,^ ^63^. The mechanism by which hospital discharge is delayed and the recovery process is affected can be explained by the complexity of the pathology that required surgical intervention and by prolonged exposure to the anesthetic and surgical procedure and anesthetic agents^22^. A study showed that an increase in the radical prostatectomy operative time of 30 or 60 minutes was associated with 1.6 and 2.8 times increased risks of symptomatic venous thromboembolic events^21^. The association between time of surgery and well-being in the present study can be explained by the participant’s (positive) cognitive evaluation of having successfully undergone the surgical and anesthetic procedure, with an expectation of cure for prostate cancer.

Surgical treatment for prostate cancer involves potential benefits and risks^3^ ^,^ ^64^ ^-^ ^65^. Factors inherent to the patient and to the surgical process may influence the development of problems related to cancer treatment and its duration. Many problems persist for years, affecting the patient’s quality of life and well-being^66^ ^-^ ^68^.

Regarding the clinical variables, pain was a predictor of quality of life in the present study. This symptom was pointed out as a common factor associated with radical prostatectomy and related to the reduction of patients’ quality of life, particularly regarding social function, walking and work activities, but the impact on these activities decreased with time^24^ ^-^ ^25^. 

In the present study, regarding the emotional variables, anxiety was a predictor of subjective well-being, as well as of quality of life. On the other hand, depression was only a predictor of quality of life. However, in the prediction of anxiety in relation to well-being, as well as depression in relation to quality of life, the results indicated a direct relation, that is, the increase in the first predictive variable was associated with an increase in the outcome variable. 

According to the literature, psychological symptoms such as anxiety and depression were related to worse postoperative outcomes and quality of life, as well as sensitivity to post surgery symptoms such as pain. These symptoms may negatively influence motivation, level of energy, coping with the disease and adherence to treatment^34^.

The emotional distress experienced by the patient with prostate cancer may be related to fear of the limitations inherent to the disease and the treatment and fear of death. Emotional stress can also be generated by distorted interpretations of reality, by real evaluations or unpleasant memories, and by pessimistic projections regarding the treatment^69^. Anxiety and depression can negatively influence motivation, energy, coping with the disease, adherence to treatment and, consequently, the patients’ well-being^34^. 

Regarding the type of coping, in the present study, problem-focused coping was a predictor of subjective well-being, whereas emotion-focused coping was a predictor of quality of life. One study pointed out that the intensity of the postoperative symptoms was inversely related to the capacity to deal with stressful situations^28^. Other study has shown that patients have tendencies to deal with situations by focusing on problems rather than focusing on emotions^30^. In this sense, problem-focused coping was a positive predictor of psychological well-being, whereas emotion-focused coping was negatively associated with well-being^29^. Patients undergoing radical prostatectomy who used problem-focused coping experienced less anxiety and depression compared to those who used emotion-focused coping^70^. Problem-focused coping was a predictor of quality of life in the six and twelve-month postoperative period of radical prostatectomy^68^.

However, in our research, emotion-focused coping was a predictor of quality of life. These results generate new points of view on ways of coping, since they are in opposition to those pointed out in the literature^30^ ^,^ ^68^.

Regarding the variable satisfaction with social support, despite its relevance in situations of chronic diseases in which social support is present, in this study, it was not a predictor of well-being or quality of life. 

Marital satisfaction, however, was a predictor of both subjective well-being and quality of life. The results showed that increases in the scores of marital satisfactions, that is, greater desire for changes in the marital relationship, were associated with increased quality of life and well-being. The type and time of the conjugal relationship may have influenced such results. The management of situations such as those faced by men who underwent radical prostatectomy may result in conjugal dissatisfaction. On the other hand, getting away from marriage demands can result in increased well-being. Marital support is reported in the literature as a predictor of quality of life^37^ ^-^ ^39^.

 In the treatment of prostate cancer, spouses take on the role of maintaining emotional balance, internalizing their feelings to try to keep a positive outlook for their partners. The responses of spouses to the results of the treatment can affect their own quality of life and the patients’^38^ ^,^ ^71^. A study pointed out that marital support was associated with higher levels of quality of life and it was essential for marital adjustment^72^ ^-^ ^73^.

Regarding the outcome variables of the present study, it is important to highlight that subjective well-being is associated with mental health aspects and, to a lesser degree, with physical variables^74^. Subjective well-being can be affected by a number of factors, such as personality characteristics, health conditions, ability to manage economic life, presence of supportive relationships, place of living, freedom to make life choices, and enjoying work activities^7^ ^-^ ^8^. In the present study (Table 2), the predictors of well-being were time of surgery, anxiety, problem-focused coping, and the desire for changes in marital satisfaction.

The distribution of means of well-being from T1 to T4 did not show differences in relation to T0. This result may be related to the observation period (360 days), which may have been insufficient to recover from the psychological effects related to frustrations and non-acceptance of changes required by the disease and treatment. Therefore, the level of well-being remained stable, unlike a study that reported that this factor remained stable in the first months after radical prostatectomy, but it increased after three months^6^. 

In the present study, as discussed above, the increase in well-being was related to greater desire to change the marital relationship. Thus, these results can be considered unusual, since the literature reports that increased well-being is related to increased marital satisfaction^38^ ^,^ ^71^. Increased anxiety also had a positive relationship with increased quality of life. On the other hand, the literature highlights that anxiety is a predictor of several undesirable outcomes after surgery. However, it was also considered a predictor of quality of life in a study of prostatectomized men^70^.

The relevance of assessing the level of well-being is supported by evidence from studies that pointed out that a high level of subjective well-being contributed to the surgical recovery process, since it increased the patient’s energy level and favored the performance of activities of daily living^6^ ^-^ ^8^. Subjective well-being was also considered a protective factor against mental illness, psychopathological symptoms and biomarkers of physical health^75^. On the other hand, low well-being negatively influenced the functional and emotional outcomes of patients in the postoperative period^76^. Negative impacts on psychological well-being and general health after radical prostatectomy were related to physiological problems derived from the surgical treatment, such as urinary incontinence and/or erectile dysfunction^77^ ^-^ ^78^.

Regarding quality of life, the other outcome of this study, it should be pointed out that in all postoperative periods the mean scores obtained were lower than those of T0, suggesting that in T4 the participants had not yet recovered the baseline condition. However, one study found that about 90% of patients reached the baseline quality of life after a mean period of five months^27^. Another study identified that quality of life three and six months after treatment was lower than the baseline, especially the results related to urinary function^79^. Authors report that the persistence of adverse effects such as sexual impotence and urinary incontinence may last for two^4^ to four years^80^, which reinforces the findings of the present study. 

Regarding the factors that may influence quality of life found in this study, pain, anxiety and depression were negative predictors of quality of life, whereas emotion-focused coping strategies and high scores on the marital satisfaction scale were positive predictors (Table 3). 

The challenges posed by prostate cancer affect not only the quality of life of the individual, but also the quality of the relationship between the patients and their spouses. Studies indicate that the general stress associated with care and concerns generated sleep disturbances and impaired well-being and quality of life of the spouse^71^. In addition, couples who used strategies to avoid or defend themselves from cancer concerns and sexual changes have dealt better with prostatectomy-related losses and transformations^39^. In this sense, the results of this research are unusual, since the desire to change the conjugal relationship, that is, conjugal dissatisfaction, had a positive association with quality of life and well-being. In addition, emotion-focused coping was positively related to quality of life, which diverges from the expected, but may represent the expectation that cognitive and behavioral efforts aimed at reducing emotional stress will result in a better quality of life.

Researches with the same characteristics explaining the positive associations between desire for change in marital relationship and well-being and quality of life were not found in the literature. These associations may be explained in new studies that consider mediating or moderating variables of this outcome, such as coping strategy, social standards, values, expectations of the spouse’s role, health conditions, among others.

The results presented reinforce some predictions described in the literature, but for other variables, the predictions are not supported by the findings of this study. Regarding these divergences, this research provides support for future research, in particular for having used valid measures, with adequate Cronbach alpha values, to obtain the data. In addition, it contributes to increase the health team’ attention on the influence of such variables on the patient’s recovery when undergoing prostatectomy. 

However, some limitations can be pointed out: the instruments were completed with the researcher reading the instructions and the items; the operationalization of the data had an important loss to follow-up; and the variation of the window for data collection, conditioned to the dynamics of the outpatient clinic or to the clinical needs of the participants.

## Conclusion

The results of this research indicate that the variables time of surgery, problem-focused coping, anxiety and desire for changes in the marital relationship were predictors of subjective well-being. The variables pain, anxiety and depression were negative predictors, whereas emotion-focused coping strategies and the desire for changes in marital satisfaction levels were positive predictors of quality of life for men who underwent radical prostatectomy in a one-year follow-up period. Thus, this research presents some prediction results distinct from those presented in the literature: marital satisfaction presented an inverse relationship with quality of life and well-being, emotion-focused coping was a predictor of quality of life and anxiety was a positive predictor of social well-being. 
